# *Pseudomonas* Evades Immune Recognition of Flagellin in Both Mammals and Plants

**DOI:** 10.1371/journal.ppat.1002206

**Published:** 2011-08-25

**Authors:** Bart W. Bardoel, Sjoerd van der Ent, Michiel J. C. Pel, Jan Tommassen, Corné M. J. Pieterse, Kok P. M. van Kessel, Jos A. G. van Strijp

**Affiliations:** 1 Medical Microbiology, University Medical Center Utrecht, Utrecht, The Netherlands; 2 Plant-Microbe Interactions, Institute of Environmental Biology, Faculty of Science, Utrecht University, Utrecht, The Netherlands; 3 Centre for BioSystems Genomics, Wageningen, The Netherlands; 4 Department of Molecular Microbiology, Institute of Biomembranes, Utrecht University, Utrecht, The Netherlands; National Institute of Allergy and Infectious Diseases, National Institutes of Health, United States of America

## Abstract

The building blocks of bacterial flagella, flagellin monomers, are potent stimulators of host innate immune systems. Recognition of flagellin monomers occurs by flagellin-specific pattern-recognition receptors, such as Toll-like receptor 5 (TLR5) in mammals and flagellin-sensitive 2 (FLS2) in plants. Activation of these immune systems via flagellin leads eventually to elimination of the bacterium from the host. In order to prevent immune activation and thus favor survival in the host, bacteria secrete many proteins that hamper such recognition. In our search for Toll like receptor (TLR) antagonists, we screened bacterial supernatants and identified alkaline protease (AprA) of *Pseudomonas aeruginosa* as a TLR5 signaling inhibitor as evidenced by a marked reduction in IL-8 production and NF-κB activation. AprA effectively degrades the TLR5 ligand monomeric flagellin, while polymeric flagellin (involved in bacterial motility) and TLR5 itself resist degradation. The natural occurring alkaline protease inhibitor AprI of *P. aeruginosa* blocked flagellin degradation by AprA. *P. aeruginosa aprA* mutants induced an over 100-fold enhanced activation of TLR5 signaling, because they fail to degrade excess monomeric flagellin in their environment. Interestingly, AprA also prevents flagellin-mediated immune responses (such as growth inhibition and callose deposition) in *Arabidopsis thaliana* plants. This was due to decreased activation of the receptor FLS2 and clearly demonstrated by delayed stomatal closure with live bacteria in plants. Thus, by degrading the ligand for TLR5 and FLS2, *P. aeruginosa* escapes recognition by the innate immune systems of both mammals and plants.

## Author Summary

*Pseudomonas aeruginosa* is a common environmental bacterium that can infect and cause disease in a wide variety of hosts, ranging from humans to plants. In healthy individuals, the innate immune system can counteract this microorganism effectively; however immunocompromised patients and cystic fibrosis patients suffer from severe infections with this bacterium. *P. aeruginosa* can propel itself through tissue by rotation of its long tail, called the flagellum, which is essential to establish colonization and infection of the host. The building blocks of the bacterial flagellum are over a thousand copies of the highly conserved protein flagellin. Mammals and plants have developed recognition systems to detect many different bacteria by sensing flagellin via Toll-like receptor 5 and Flagellin-sensitive 2, respectively. Bacteria actively try to interfere with this recognition (immune evasion). In this study, we describe a novel mechanism of *P. aeruginosa* to escape flagellin recognition. The secreted protein alkaline protease of *P.* aeruginosa, degrades immunity activating free flagellin. Bacterial motility is maintained, because flagellin present as building block of flagella is not degraded. In this way, the bacterium impairs recognition and hides itself from destruction by the immune system. Understanding these immune evasion strategies is of extreme importance for the development of new therapeutic approaches.

## Introduction

The innate immune system detects microorganisms and rapidly responds to invasion by eliminating them. Toll-like receptors (TLRs) recognize various evolutionary conserved structures of microorganisms and play a crucial role in innate immune recognition [1]. Stimulation of these receptors triggers intracellular signaling cascades leading to activation of phagocytes and production of pro-inflammatory cytokines. TLRs are type-1 transmembrane proteins characterized by extracellular leucine-rich-repeat motifs and an intracellular Toll/interleukin-1 receptor domain. Dimerization of TLRs is important for activation and ligand recognition, for example TLR2 recognizes diacylated lipopeptides in combination with TLR1 and triacylated lipopeptides together with TLR6. The most studied TLR member is TLR4, which detects the Gram-negative outer membrane component lipopolysaccharide (LPS). TLR5 senses flagellin [2], which is the major component of the bacterial flagellum.

Flagella consist of a basal body, the flagellar hook and a filament which serves as a propeller [3]. The filament consists of 11 protofilaments composed of several thousand flagellin monomers. Flagellin molecules from various bacteria have a conserved N- and C-terminus and a hypervariable central domain. The conserved regions are important in protofilament formation and motility. TLR5 recognizes a conserved part of flagellin that is buried in the flagellar filament and is only accessible in flagellin monomers [4]. By recognizing this part of flagellin, TLR5 detects almost all flagellated bacteria. Mutation of the TLR5-recognition site generally impairs protofilament assembly and thereby motility and virulence [5]. However, in the human pathogens *Campylobacter jejuni* and *Helicobacter pylori* the flagellin is changed in such a way that it is no longer recognized by TLR5, while motility is not affected [6].

Plants have evolved a similar sensing system for flagellin as mammals [7]. In *Arabidopsis thaliana*, stimulation of the plasma membrane-located receptor FLS2 [8] results in the activation of defense responses, such as callose deposition and the production of pathogenesis-related proteins [7]. Flagellin recognition contributes to the resistance of *Arabidopsis* to the bacterial pathogen *Pseudomonas syringae* [9]. Although TLR5 and FLS2 serve a similar function in pathogen recognition, the composition of the receptor, as well as the downstream signaling pathways differ considerably. Furthermore, FLS2 recognizes a different epitope of flagellin than does TLR5. A peptide, called flg22, consisting of 22 amino acids derived from the highly conserved N-terminal region of *P. syringae* flagellin activates FLS2 even better than purified flagellin [7], [10].

*Pseudomonas aeruginosa* is a common environmental Gram-negative bacterium, which acts as an opportunistic pathogen in humans and plants. Normally, the human host counteracts this microorganism effectively via the innate immune system [11]. However, immunocompromised patients, severe burn victims and cystic fibrosis patients are sensitive for *P. aeruginosa* infections. Due to its tendency to colonize surfaces in a biofilm, the bacterium is impervious to therapeutic concentrations of many antibiotics [12].

Detection of *P. aeruginosa* by TLRs activates the innate immune system and protects the host from infection [13]. Flagellin of *P. aeruginosa* is a potent TLR5 activator. It is released during bacterial growth, because the long flagellum tail is easily disrupted [14]. The contribution of TLR5 to the inflammatory response of *P. aeruginosa* may be masked by activation of TLR4 by bacterial LPS [15]. Both receptors cooperate to defend the host from infection: the absence of both TLR4 and TLR5 results in hypersusceptibility for lung infection in mice [16]. Recognition of *P. aeruginosa* flagellin is important for the efficient clearance of the bacterium in mice [17].

Two extracellular proteases of *P. aeruginosa* that exert their activity at the invasive stage have been associated with virulence i.e. elastase and alkaline protease. Elastase cleaves collagen, IgG, IgA, and some proteins of the complement system [18]. It also degrades fibronectin to expose receptors for bacterial attachment on the mucosa of the lung [19]. Elastase disrupts the respiratory epithelium and interferes with ciliary function. So far, alkaline protease and elastase together are also reported to cause the inactivation of gamma interferon (IFN-γ) and tumor necrosis factor alpha (TNF-α) [20]. A *Pseudomonas entomophila* strain, that lacks alkaline protease was shown to be less virulent and persistent in a *Drosophila* infection model [21].

Immune evasion is an important strategy for bacteria to survive in their host. Immune evasion molecules are crucial for this survival, as has been demonstrated for several human and plant pathogens [22], [23], [24], [25]. During infection, monomeric flagellin released from bacterial flagella activates TLR5 signaling. To evade innate immune recognition, some bacteria have evolved strategies in which they manipulate flagellin to impair TLR5 activation [6]. We hypothesized that bacteria secrete proteins that interfere with recognition of TLRs. In our search for immune evasion molecules that act as TLRs antagonists, we identified alkaline protease of *P. aeruginosa* as a TLR5 signaling inhibitor.

## Results

### A secreted protein of *P. aeruginosa* inhibits TLR5 activation

**Figure 1:**
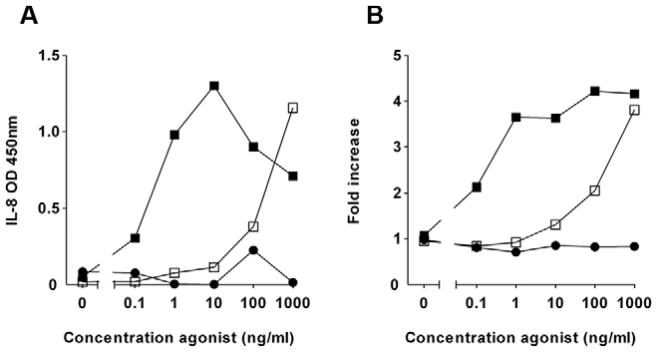
Fractionated *P. aeruginosa* supernatant inhibits TLR5 activation. HEK/TLR5 cells were transfected with a NF-κB reporter construct. Cells were incubated with 20-fold diluted elution fraction from a Q sepharose column (inhibitor) for 30 min and subsequently challenged with various concentrations recombinant flagellin of *S.* Typhimurium (□). Flagellin without inhibitor (▪) and LPS (•). (A) After 6 h the IL-8 concentration in the cell culture supernatant was measured by ELISA. (B) NF-κB activation was determined by measuring luciferase activity in a luminometer and expressed as fold increase of luciferase activity over stimulation with culture medium alone. The presented data are representative for the inhibition of TLR5 signaling that is typically observed with purifications of *P. aeruginosa* supernatant.

To identify TLR5 inhibitors, we screened several culture supernatants of Gram-positive and Gram-negative bacteria for inhibitory activity on activation of TLR5-transfected HEK cells (HEK/TLR5) containing an NF-κB luciferase reporter. Stimulation of HEK/TLR5 with flagellin triggers the activation of NF-κB and the secretion of Interleukin 8 (IL-8) into culture supernatants. These cells are insensitive for the TLR4 ligand LPS, whereas they respond to monomeric flagellin in the picomolar range (Figure 1A and B). Most bacterial supernatants did not significantly inhibit IL-8 production upon flagellin stimulation. However, the supernatant of *P. aeruginosa* reduced activation of HEK/TLR5 cells consistently with about 30%. Therefore, this supernatant was chosen to isolate the potential TLR5 inhibitor.

To purify the inhibitory compound from *P. aeruginosa*, we fractionated the supernatant with ion-exchange chromatography. In an agonist dose response experiment, specifically eluted fractions inhibited IL-8 production completely when the HEK/TLR5 cells were stimulated with up to 10 ng/ml flagellin of *Salmonella enterica* serovar Typhimurium (*S.* Typhimurium) (Figure 1A). In a parallel independent assay, the same fractions inhibited flagellin-stimulated NF-κB activation (Figure 1B). LPS-stimulation of HEK/TLR4 cells was not affected by the partially purified *P. aeruginosa* supernatant (Figure S1), which suggests that the inhibitor acts at the receptor level and not at downstream signaling routes. Additional purification of the inhibitory activity by size-exclusion chromatography resulted in one band by SDS-PAGE that correlated with TLR5-inhibiting activity of eluted fractions (Figure S2). We identified, using mass-spectrometry, the protein of interest as alkaline protease.

### AprA inhibits TLR5 activation

**Figure 2:**
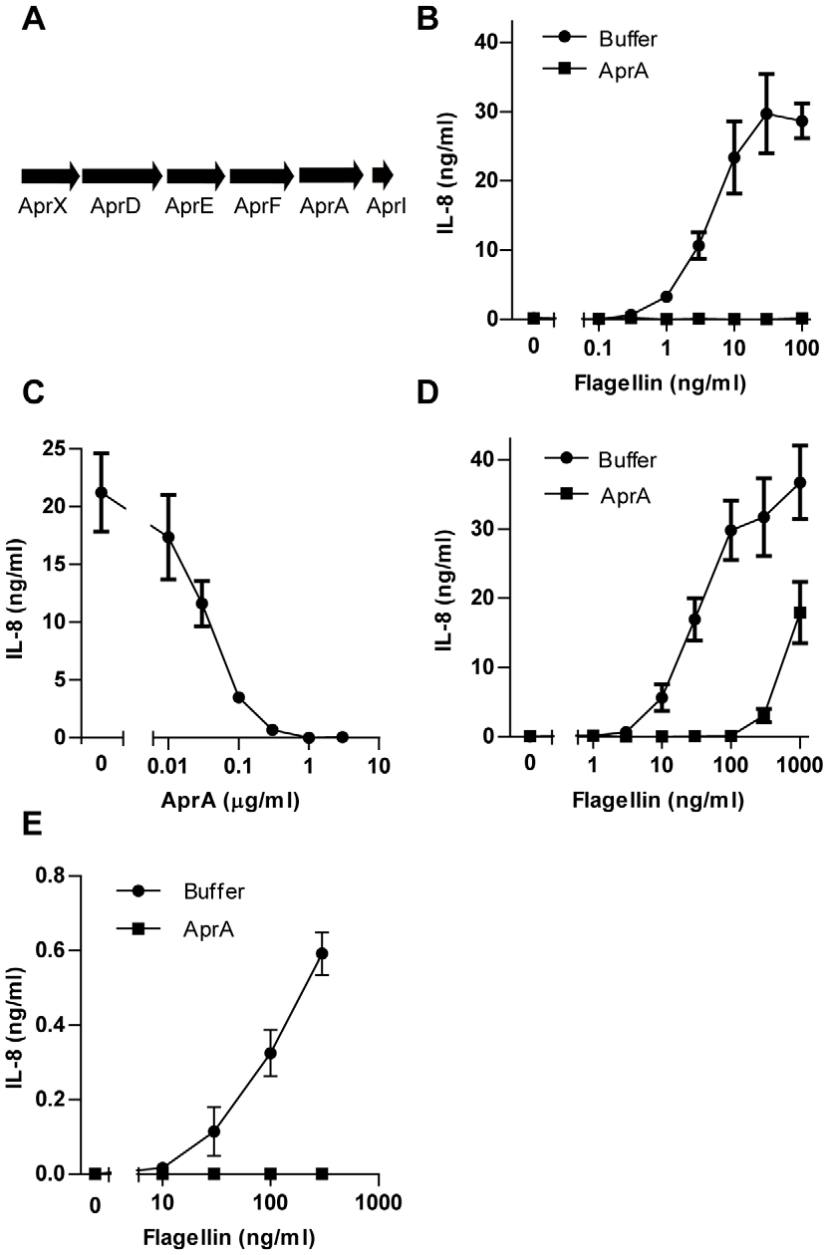
AprA prevents flagellin-induced IL-8 production by HEK/TLR5 cells. (A) Schematic representation of the gene cluster of *aprA*, *aprI*, genes involved in secretion, *aprD*, *aprE*, and *aprF* and *aprX* on the genome of *P. aeruginosa* strain PAO1^26^. (B) Different concentrations of recombinant flagellin from *S.* Typhimurium were treated with 1 µg/ml recombinant His-AprA for 30 min and subsequently added to HEK/TLR5 cells. After 6 h IL-8 was measured in the supernatant by ELISA. (C) His-AprA concentration-dependent inhibition of flagellin-induced HEK/TLR5 cell activation. HEK/TLR5 cells were treated with varying concentrations of AprA and challenged with 30 ng/ml flagellin of *P. aeruginosa.* (D) Recombinant flagellin of *P. aeruginosa* was incubated with buffer or 1 µg/ml His-AprA for 30 min and subsequently added to HEK/TLR5 cells for IL-8 release. (E) Recombinant flagellin of *P. aeruginosa* was incubated with 1 µg/ml recombinant His-AprA in the presence of PMB (10 µg/ml) for 30 min at 37°C, and subsequently added to human neutrophils. After 16 h IL-8 concentration was measured by ELISA. Results represent mean IL-8 concentration ± SEM from three independent experiments.

The gene *aprA* of *P. aeruginosa* encodes alkaline protease (designated AprA), which is a 50 kD zinc metalloprotease [26]. AprA is secreted via a type I secretion system, which is encoded by the genes a*prD*, *aprE* and *aprF* [27] (Figure 2A). The gene downstream of *aprA*, a*prI*, encodes a highly specific inhibitor of AprA [28]. Some biological substrates of AprA have been described, such as the cytokine IFN-γ [20]. To verify that AprA inhibits TLR5-mediated cell activation, we cloned and expressed *aprA* with a 6x His-tag or together with the genes encoding the secretion apparatus in *Escherichia coli* and purified AprA from the medium. The TLR5 inhibitory activity of recombinant His-AprA and secreted recombinant AprA (data not shown) was comparable to that of purified AprA from *P. aeruginosa* (Figure 1A and 2B). Incubation of HEK/TLR5 cells with recombinant AprA abolished IL-8 production completely even when the cells were stimulated with flagellin concentrations up to 100 ng/ml (Figure 2B). To determine the potency of AprA, different concentrations were tested for inhibition of the flagellin-induced IL-8 production by HEK/TLR5 cells. Complete inhibition of cell activation by 30 ng/ml *P. aeruginosa* flagellin was observed with 0.3 µg/ml AprA with a half maximal inhibitory concentration of 30 ng/ml (Figure 2C). Flagellin isolated from *P. aeruginosa* also served as a potent stimulator of HEK/TLR5 cells. As for *S.* Typhimurium flagellin, AprA completely inhibited the *P. aeruginosa* flagellin response (Figure 2D). HEK/TLR5 cells are unresponsive to lipopolysaccharide, a contaminant of recombinant proteins isolated from *E. coli*, in contrast to naturally TLR5 sufficient cells like human neutrophils. To investigate the effect of AprA on TLR5 activation of human neutrophils, we incubated *P. aeruginosa* flagellin with AprA in the presence of polymyxin B, which neutralizes LPS activity. In addition to HEK/TLR5 cells, treatment of *P. aeruginosa* flagellin with 1 µg/ml AprA inhibited the IL-8 production of neutrophils completely (Figure 2E). These data demonstrate that AprA from *P. aeruginosa* is an inhibitor of TLR5-mediated cell activation by different types of flagellin.

### AprA cleaves flagellin

**Figure 3:**
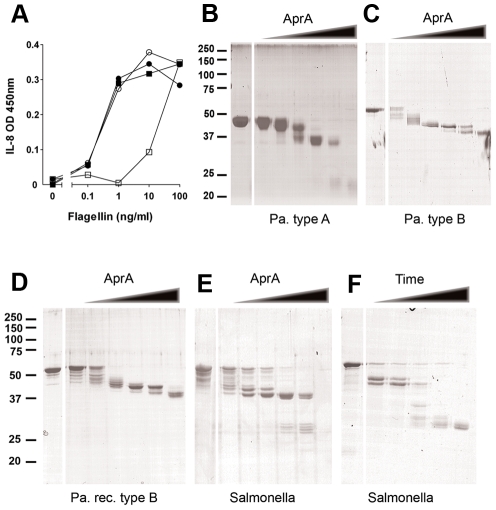
AprA of *P. aeruginosa* cleaves flagellin. (A) HEK/TLR5 cells were treated for 30 min with AprA purified from *P. aeruginosa* culture supernatant and directly stimulated (□) or washed (○) before stimulation with varying concentrations flagellin from *S.* Typhimurium. As control cells were directly stimulated (•) or washed (▪) before addition of flagellin. After 6 h incubation, IL-8 was measured in the supernatant of HEK/TLR5 cells by ELISA. (B–F) Degradation of flagellin by recombinant AprA. Flagellin was mixed with 0, 0.01, 0.03, 0.1, 0.3, 1 and 3 µg/ml AprA for 60 min at 37°C in PBS and protein degradation was analyzed by SDS-PAGE and Coomassie staining. Cleavage of flagellin by AprA was compared for native monomeric (B) flagellin type B isolated from *P. aeruginosa* strain PAO25 (1 mg/ml), (C) flagellin type A isolated from clinical *P. aeruginosa* strain (150 µg/ml), (D) recombinant flagellin type B from *P. aeruginosa* (250 µg/ml) and (E) recombinant flagellin of *S.* Typhimurium (250 µg/ml). (F) Time-dependent degradation of flagellin by AprA. Flagellin (250 µg/ml) of *S.* Typhimurium was incubated with AprA for 0, 1, 3, 10, 30 and 60 min at 37°C in PBS.

To determine the mechanism of TLR5 inhibition, we investigated the effect of AprA on TLR5 and flagellin separately. To study any direct effect of AprA on TLR5, cells were incubated with the protease for 30 min, washed and subsequently stimulated with flagellin. A washing step between AprA incubation and addition of flagellin preserved TLR5 activation (Figure 3A). This result indicates that TLR5 is not affected by AprA and still properly responds to flagellin.

Another possible mechanism to interfere with TLR5 recognition is neutralization or proteolysis of flagellin. To test this hypothesis, isolated flagellin was incubated with AprA and degradation was analyzed by SDS-PAGE. Flagella from *P. aeruginosa* are composed of either type A or B flagellin, depending on the strain. These two flagellins contain a completely different variable domain, but showed comparable degradation patterns upon incubation with increasing concentrations AprA (Figure 3B and C). An identical cleavage pattern was observed for recombinant *P. aeruginosa* flagellin type B (Figure 3D). Moreover, AprA cleaved flagellin from another species i.e. *S.* Typhimurium (Figure 3E). Degradation of flagellin was time-dependent and started within one minute after addition of AprA (Figure 3F). Cleavage of flagellin occurred in multiple steps, dependent on the protease concentration and incubation time. At higher concentrations of AprA, flagellin type A (Figure 3B) and *S.* Typhimurium (Figure 3E) flagellin were completely degraded, while for *P. aeruginosa* flagellin type B (Figure 3C and D) a truncated protein of about 37 kD remained visible. The proteolytic activity of AprA is inhibited by 100 mM EDTA [29]. In our experiments EDTA also abolished degradation of flagellin (Figure S3). The optimum pH for AprA is pH 9–10 [18]; however in our experiments flagellin was efficiently degraded under physiological conditions. These findings demonstrate that *P. aeruginosa* secretes a protease that efficiently degrades flagellin of different species and thereby prevents activation via TLR5.

### Flagellar filaments are not degraded by AprA

**Figure 4:**
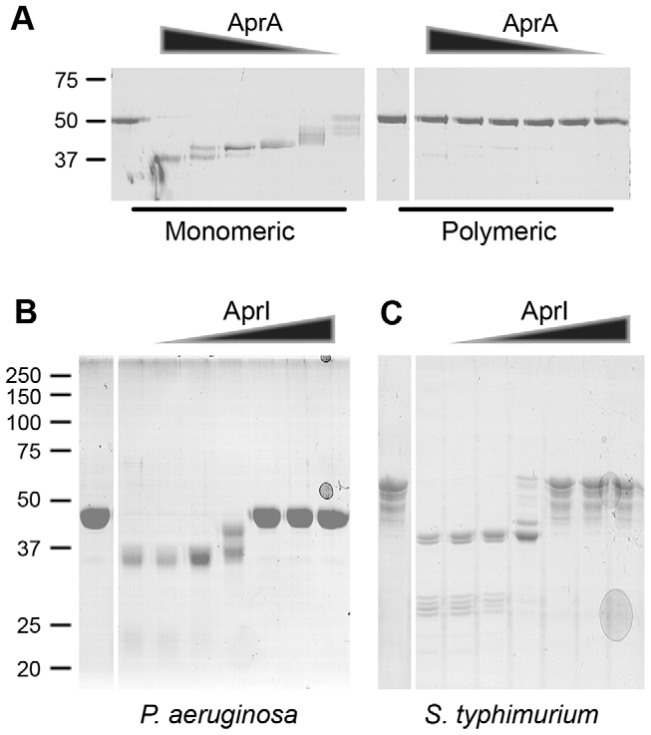
Polymeric flagellin resists cleavage by AprA and AprI is an efficient endogenous inhibitor. (A) Flagella isolated from PAO1 were treated for 20 min at 70°C to obtain monomeric flagellin. Untreated polymeric flagellin was compared with monomeric flagellin for susceptibility to AprA cleavage. Monomeric and polymeric flagellin was incubated with 0, 3, 1, 0.3, 0.1, 0.03 or 0.01 µg/ml AprA for 60 min at 37°C and analyzed by SDS-PAGE. (B and C) AprA (1 µg/ml) was incubated with 0, 0.03, 0.1, 0.3, 1, 3 or 10 µg/ml AprI and subsequently flagellin of (B) *P. aeruginosa* or (C) *S.* Typhimurium was added. Samples were analyzed by SDS-PAGE, untreated flagellin control is shown in the first lane followed by increasing AprI concentrations.

Flagellin is the most abundant protein of the flagellum, which is essential for bacterial motility and virulence. Secretion of a protease by *P. aeruginosa* that degrades the flagellum would be disadvantageous for the bacterium. Therefore, we investigated whether AprA also degrades complete flagellar filaments isolated from *P. aeruginosa*. These filaments consist of polymerized flagellin. Incubation of flagellin polymers with AprA did not result in degradation of flagellin (Figure 4A). However, after depolymerization, the resulting monomeric flagellin was degraded by AprA. Even at higher concentrations AprA did not cleave flagellar filaments as observed for monomeric flagellin. This indicates that AprA inactivates only monomeric flagellin in the surrounding of the bacterium, while the integrity of flagella is preserved.

### AprI blocks AprA-mediated cleavage

Downstream of the *aprA* gene, *aprI* is located which, encodes for alkaline protease inhibitor (designated AprI). Kinetic studies revealed a very high affinity of this 11.5 kD protein for AprA [28]. To investigate whether AprI interferes with AprA-mediated cleavage, we cloned and expressed the *aprI* gene in *E. coli* as a His-tagged protein and purified it using Ni-affinity chromatography. AprI prevented cleavage of flagellin slightly at equimolar concentrations (Figure 4B and C). Flagellin cleavage was completely blocked at higher AprI concentrations. AprI protected both *P. aeruginosa* and *S.* Typhimurium flagellin against AprA-mediated proteolysis. Importantly, incubation of AprA with AprI before addition of flagellin restored activation of HEK/TLR5 cells dose-dependently (Figure S4). In conclusion, AprI of *P. aeruginosa* is a very potent inhibitor of AprA-mediated cleavage of flagellin.

### *Pseudomonas aprA* mutant activates TLR5

**Figure 5:**
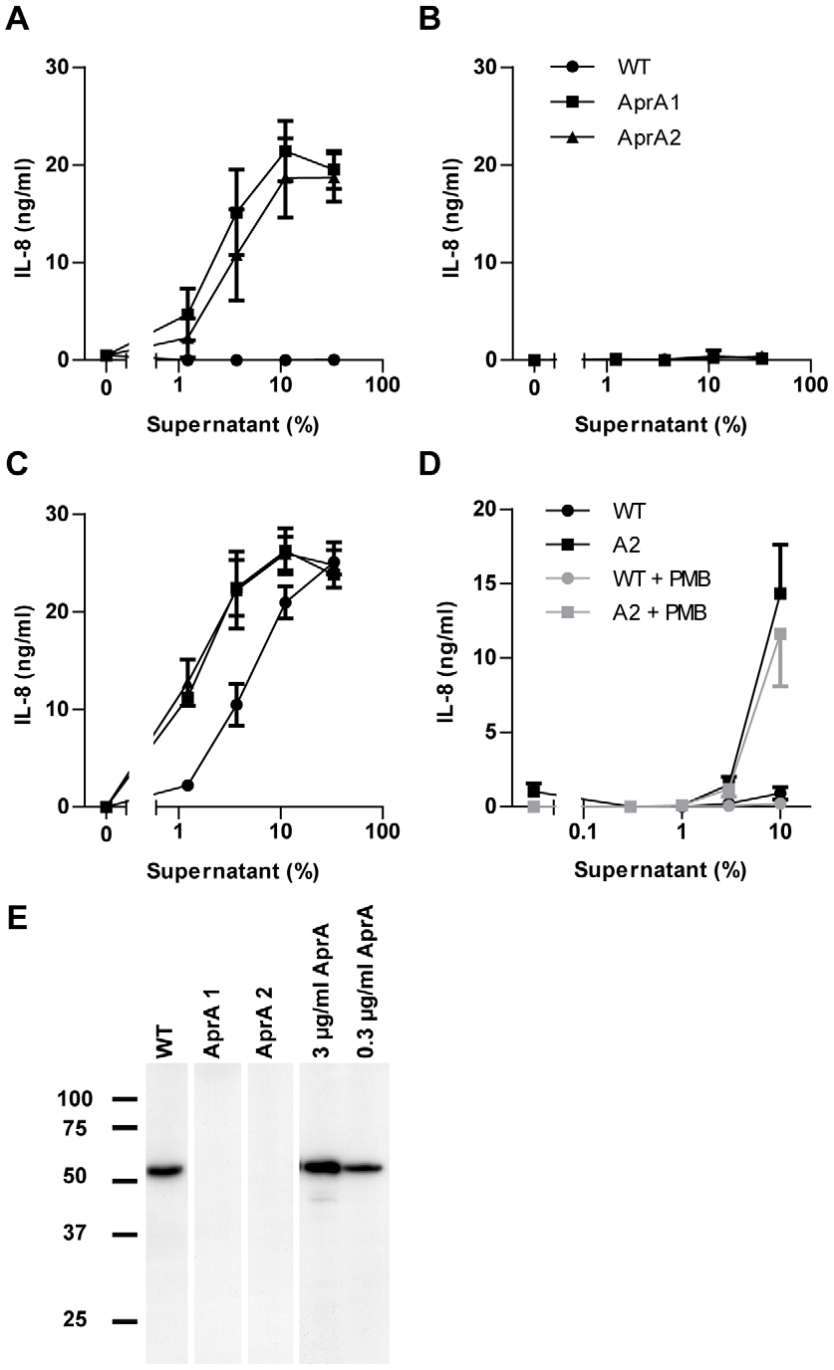
Culture supernatant of *aprA* mutant strains trigger TLR5. (A) Dilutions of bacterial culture supernatants, collected from overnight grown wild-type (WT), and isogenic *aprA* mutant strains were used as flagellin source to stimulate HEK/TLR5 cells for IL-8 production. (B) 3 µg/ml recombinant AprA was added to the culture medium before inoculation with WT, *aprA*1 or *aprA*2 mutant strains. HEK/TLR5 cells were incubated with dilutions of bacterial culture supernatants and stimulated for 6 h. IL-8 production was measured by ELISA. All three data sets completely overlap in this graph. (C) Wild-type and mutant *P. aeruginosa* strains were grown in the presence of 10 µg/ml exogenous AprI and dilutions of the culture supernatants were added to HEK/TLR5 cells for IL-8 release. Data are expressed as mean IL-8 concentration ± SD from triplicates. (D) Human neutrophils were stimulated with dilutions of bacterial culture supernatants of wild-type and *aprA*2 mutant strain. PMB (10 µg/ml) was added prior to stimulation for 30 min at 37°C. After 16 h stimulation, IL-8 concentration in cell supernatant was determined. Results represent mean ± SEM of three independent experiments. (E) Culture supernatant of overnight grown wild-type and *aprA* mutant strains or recombinant AprA were analyzed for the presence of AprA by immunoblotting.

As demonstrated above, purified recombinant AprA effectively blocked flagellin-induced TLR5 activation. To address the importance of this protease in a more natural environment, we compared the TLR5-activating capacity of culture supernatants from *P. aeruginosa* wild-type (WT) and *aprA* transposon-insertion mutants. In this setup, we tried to understand the dynamic interaction between the endogenous flagellin and AprA when released simultaneously by growing bacteria. Only the supernatant of the *aprA* mutant strains triggered TLR5 signaling (Figure 5A). Strikingly the supernatant of the wild-type strain did not initiate IL-8 production at all. This experiment indicates that AprA completely degraded flagellin monomers that were released in the supernatant during overnight growth. As expected, dilution of supernatants of the *aprA* mutants limited TLR5 activation due to a decrease in flagellin concentration (Figure 5A). To show that the absence of AprA is responsible for the TLR5-activating capacity in the *aprA* mutant strains, we supplemented the culture medium of these strains with recombinant AprA before inoculation. This resulted in the same lack of activation as for the wild-type strain supernatant (Figure 5B), demonstrating that AprA is essential as well as sufficient for degradation of flagellin. Complementary, addition of AprI to the culture medium abolished AprA-mediated cleavage of flagellin in the wild-type strain resulting in activation of TLR5 to comparable levels of that of the *aprA* mutant strains (Figure 5C). These results show that the wild-type strain does release flagellin in its environment. To investigate the inflammatory response of naturally TLR4 and TLR5 sufficient cells, we stimulated neutrophils with bacterial supernatant of wild-type and an *aprA* mutant strain. As for HEK-TLR5 cells, the aprA mutant strain triggered higher IL-8 production in comparison to wild-type *P. aeruginosa* (Figure 5D). Addition of polymyxin B, to neutralize LPS, slightly inhibited IL-8 production of neutrophils in response to supernatant of wild-type and the *aprA* mutant strain, without changing the difference in IL-8 production between the two strains. To verify that the *aprA* mutant strains did not produce AprA, the culture supernatant was probed with a specific antiserum against AprA by western blotting. As expected, the wild-type strain secreted AprA while the two *aprA* mutant strains did not (Figure 5E). Growth and motility of the mutant strains was comparable with wild-type. Together, these results demonstrate that *P. aeruginosa* secretes sufficient AprA to neutralize its own monomeric flagellin for detection via TLR5.

### AprA interferes with flagellin recognition by *Arabidopsis*

**Figure 6:**
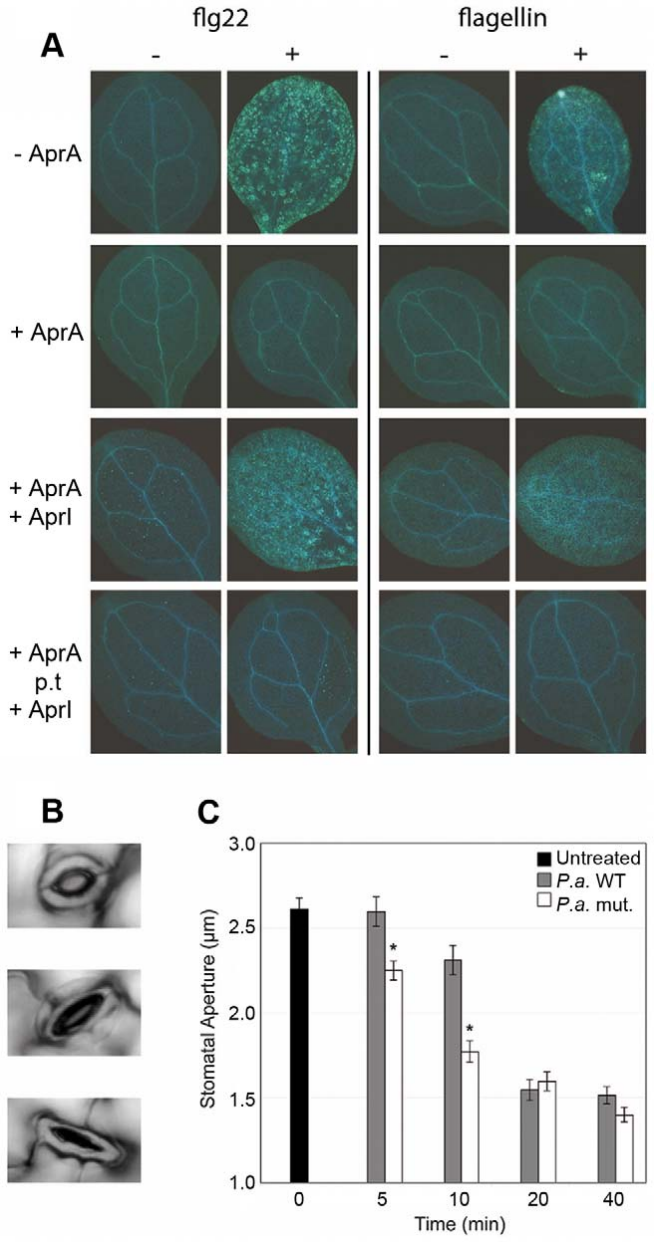
AprA prevents recognition of flagellin in *Arabidopsis* and prevents stomatal closure. *A. thaliana* La-*er* seedlings were incubated with or without 500 nM flg22 or *P. aeruginosa* flagellin preincubated with 3 µg/ml AprA when indicated. (A). After treatment for 24 h, seedlings were stained for callose deposition by aniline blue and fluorescence was photographed under UV light. In the 3^rd^ row panels AprI was added before AprA treatment and in the bottom panels post AprA treatment (p.t.) of flagellin. (B) Examples of open (top), half open (middle) and closed (bottom) stomata, that were observed during the experiment. (C) Stomatal aperture on leaves of 5-week-old *A. thaliana* plants up to 40 minutes after treatment with *P. aeruginosa* PAO1 or isogenic *aprA* mutant strain (n = 108 to 224). Error bars indicate SEM. Asterisks indicate significant differences (Student's t-test; p<0.001) between WT and AprA mutant treated plants.

Like mammals, plants also possess an innate immune system in which the detection of flagellin monomers results in the activation of effective immune responses [30]. In *Arabidopsis*, the FLS2 receptor has been demonstrated to specifically interact with a conserved 22-amino acid motif (flg22) of flagellin monomers [31]. Upon interaction of FLS2 with flagellin monomers or flg22, several downstream defense mechanisms are activated, amongst which the deposition of callose polymers [10]. Furthermore, as a result of the activation of energy-costly defense mechanisms, treatment with flagellin or flg22 has a negative effect on *Arabidopsis* growth [10]. To assess whether AprA activity can also prevent flagellin-induced defense activation in plants, we studied the effect of AprA on flagellin- or flg22-induced callose deposition and growth inhibition of *Arabidopsis* seedlings. *P. aeruginosa* flagellin monomers triggered callose deposition (Figure 6A) and affected growth (Figure S6) to a slightly less extent than did flg22, which is in line with earlier observations [30]. Preincubation of flagellin or flg22 with AprA abolished callose deposition (Figure 6A) and restored plant growth to control levels (Figure S5). This result indicates that AprA disrupts the active epitope of flagellin that is normally recognized by FLS2. Moreover, AprA completely neutralized the effects of the peptide flg22, these results suggest that AprA cleaves flagellin at least within this 22-amino acids long conserved motif. Therefore, we examined the mass of protease-treated flg22 and indeed observed clear degradation of the peptide by SELDI-TOF (Figure S6). Addition of AprI prior to AprA treatment of flagellin neutralized the AprA-mediated effects in *Arabidopsis*, while AprI had no effect when added after AprA treatment of flagellin (Figure 6A, Figure S5).

### AprA delays early plant immune responses

The epidermis of plant leaves contains many pores (stomata) of which the aperture is dependent on environmental factors such as humidity and CO_2_ concentration [32]. Previously, Melotto et al. [33] showed that besides these environmental cues, recognition of bacterial PAMPs, such as flagellin and LPS, trigger plant immune responses that lead to rapid stomatal closure (Figure 6B). In this way, the stomata have an important early defense function that actively prevents bacteria from entering the host [33]. We hypothesized that degradation of flagellin by AprA likely affects the speed of stomatal closure upon bacterial inoculation. Therefore, we monitored the stomatal aperture of *A. thaliana* leaves after inoculation with wild-type and mutant *P. aeruginosa* bacteria. Figure 6C shows that both wild-type PAO1 and the *aprA* mutant strain triggered closure of the stomata within 40 minutes. However, the stomatal aperture decreased significantly faster after treatment with the *aprA* mutant strain. Hence, we conclude that AprA produced by wild-type bacteria plays an important role in the evasion of host immunity of *A. thaliana* by hampering closure of the natural pores that are crucial for bacterial invasion.

## Discussion

*P. aeruginosa* produces various proteases that degrade several host proteins and are associated with virulence [20]. Alkaline protease of *P. aeruginosa* is involved in suppression of the immune response by degradation of cytokines including TNF-α and IFN-γ. Screening for bacterial TLR5 inhibitors resulted in the identification of AprA of *P. aeruginosa*. In this study, we describe flagellin as an AprA substrate. TLR5 is unaffected by the proteolytic activity of this metalloprotease, however its ligand flagellin is degraded effectively and loses stimulatory activity

**Figure 7:**
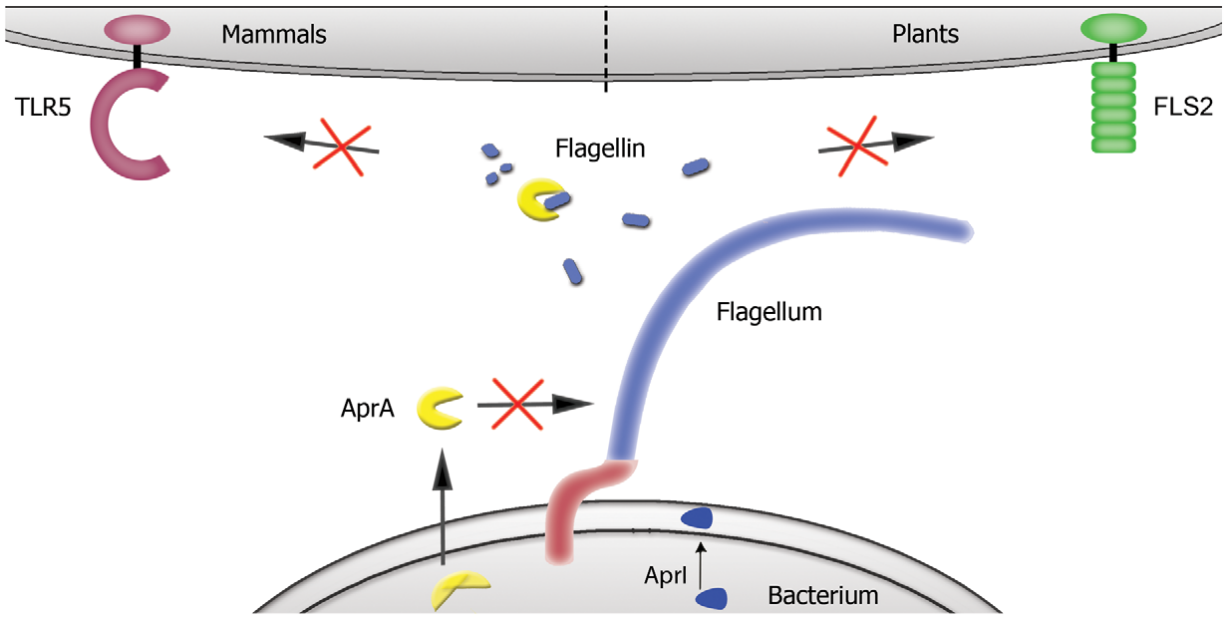
Proposed mechanism for AprA. *P. aeruginosa* secretes AprA, which degrades free monomeric flagellin in the surrounding of the bacterium, whereas polymeric flagellin present in flagella is not affected. In this way flagellin is not recognized by TLR5 and FLS2, and thereby *P. aeruginosa* escapes activation of the innate immune system in both mammals and plants. AprA is secreted in one step over both membranes and is not present in its active form in the cytoplasm of the bacterium.

The only known ligand for TLR5 is bacterial flagellin, which triggers the production of pro-inflammatory cytokines. Only monomeric flagellin activates TLR5 signaling, while in the polymeric form, which is present in flagella, the TLR5 recognition site is inaccessible. Previous studies showed that the N- and C-terminus of flagellin are located inside the flagellar filament [34]. This highly conserved part of flagellin activates TLR5 and is essential for filament assembly. Here we show that AprA fails to degrade polymeric flagellin and therefore does not affect motility. In this way, *P. aeruginosa* protects its functional flagellin present in flagella, while it neutralizes TLR5-activating monomeric flagellin (Figure 7). The proteolytic activity of AprA can be inhibited with EDTA [29] and with a natural inhibitor of *P. aeruginosa*, AprI, which blocks the catalytic site of AprA [26]. We demonstrate that AprI prevents AprA-mediated flagellin cleavage. The physiological role for AprI is unclear. An obvious possibility is that it protects intracellular bacterial proteins from degradation.

Bacterial release of monomeric flagellin [14] results in the activation of TLR5. Since picomolar concentrations of flagellin can trigger TLR5, highly efficient degradation is a prerequisite to abolish TLR5 signaling. *P. aeruginosa* produces sufficient amounts of AprA to degrade its own released flagellin completely, resulting in avoidance of TLR5 activation. Mutant *aprA* strains of *P. aeruginosa* lacking AprA illustrate that AprA is responsible for this effect, because supernatants of these strains activate TLR5 signaling. This is best demonstrated in our experiments where we use supernatants of wild-type bacteria that contain endogenous amounts of flagellin and AprA (Figure 5A). No TLR5 activation was observed in these experiments, while the same experiments with mutant strains lacking AprA showed high flagellin-mediated stimulatory capacity. The effect of TLR4 and TLR5 in the host defense against *P. aeruginosa* was shown to be redundant [13], [16]. However, TLR5 is the only TLR that is significantly higher expressed on neutrophils in the cystic fibrosis lung [35]. In addition, flagellin triggers phagocytosis of *P. aeruginosa* and oxidative burst by peripheral blood neutrophils. Supernatant of an *aprA* mutant enhanced the inflammatory response of human neutrophils in comparison to wild-type *P. aeruginosa* supernatant. This suggests that flagellin-mediated TLR5 activation is important to stimulate an inflammatory response against *P. aeruginosa*, which is inhibited by AprA. Liehl et al. [21] recently reported that an *aprA* knockout strain of *Pseudomonas entomophila* showed decreased virulence in a *Drosophila* infection model. In their model, *P. entomophila* AprA is necessary to persist in the host and for pathogenicity. Moreover, AprA protected against the *Drosophila* immune response. Although no homolog of TLR5 is described in *Drosophila*, flagellin does trigger the production of antimicrobial peptides [36]. It is tempting to speculate that the immune evasion strategy of *P. aeruginosa* is also operational in this model. In this scenario, escape of innate immune detection by AprA-mediated degradation of pathogen-derived monomeric flagellin in the environment, avoids the production of antimicrobial peptides by the host, which results in a more persistent infection with *P. entomophila*.

In plants we observe the same phenomenon. The FLS2 flagellin receptor in *Arabidopsis* recognizes a different epitope of flagellin then does TLR5. By affecting the ligand instead of targeting the receptor, *P. aeruginosa* evades recognition of flagellin by both FLS2 and TLR5. The same was true for the flagellin-derived peptide flg22, which stimulates FLS2. Stomatal closure is important to prevent bacteria from entering the plant and recognition of flagellin by FLS2 plays a profound role in this response [37]. *P. aeruginosa* lacking a functional *aprA* gene triggers faster closure of stomata in comparison with wild-type. Thus, AprA interferes with this early defense response. Although *P. aeruginosa* itself is not a true plant pathogen, other *Pseudomonas* species that infect *Arabidopsis* like *P. syringae* also contain an *apr* operon. AprA and its inhibitor AprI of *P. syringae* share similarity with AprA and AprI of *P. aeruginosa* of 71% and 50%, respectively. Hence, evasion of TLR5 and FLS2 recognition as we observed for *P. aeruginosa* may be a broad mechanism utilized by various bacterial species.

AprA belongs to the superfamily of metzincin metalloproteases and to the family of serralysins [38]. Proteases from *Serratia marcescens* and *Erwinia chrysanthemi* belong to the family of serralysins and share high sequence similarity with AprA [39]. These bacteria also possess a highly specific protease inhibitor and a similar secretion system for AprA. In these flagellated bacteria AprA homologs may degrade flagellin in the same way as observed for *P. aeruginosa*. AprA cleaves flagellin from both *P. aeruginosa* and *S.* Typhimurium. Since the cleavage site of AprA is within the conserved domain of flagellin, cleavage of flagellin from other flagellated bacteria can be expected.

Innate immune defense systems recognize evolutionary conserved structures. Evasion of immune receptors is a smart strategy of pathogens to escape activation of the host innate immune response [40], [41]. For a bacterium with a broad host range it is of great advantage to evade activation of the innate immune system by degrading the ligand that is recognized by the host. Here we provide evidence that *P. aeruginosa* has evolved such a system to circumvent TLR5 and FLS2 activation by flagellin degradation. The consequence is that the benefit for the bacterium, in this case intact flagella and movement should be protected. By secreting AprA, which is specific for monomeric form of flagellin, *Pseudomonas* has tackled this, without affecting the structure of flagellin in the flagellum. In this way *Pseudomonas* can evade the activation of pattern-recognition receptors such as TLR5 and FLS2 signaling, creating a window of opportunity to evade the immune system of different hosts and cause disease.

## Materials and Methods

### Cell culture and bacterial strains

Dulbecco's modified Eagle's medium (DMEM) and Iscoves modified Dulbecco's medium (IMDM) (Invitrogen), fetal bovine serum (FCS) (Gibco), Human embryonic kidney cells transfected with TLR4/CD14/MD-2 (HEK/TLR4) or TLR5 (HEK/TLR5) and Normocin and Blasticidin (all Invivogen) were used for cell and bacterial culture. Human neutrophils from healthy volunteers were isolated as described [40]. IL-8 ELISA kit and high performance ELISA buffer (HPE) were purchased from Sanquin. *P. aeruginosa* strain PAO25 is a *leu arg* mutant derivative of strain PAO1 [42]. Clinical isolates of *P. aeruginosa*, *S.* Typhimurium, *Staphylococcus haemolyticus*, *Staphylococcus hominis, Staphylococcus warneri, Streptococcus milleri*, *Enterobacter cloacae*, *Klebsiella pneumoniae*, *Escherichia coli*, *Serratia adorigen*, *Listeria monocytogenes* and *Enterobacter cloacae* were obtained within the UMC Utrecht and screened for TLR5 antagonists. Competent *E. coli* TOP10F' and BL21 (DE3) pLys were purchased from Invitrogen and flg22 from Genscript.

### HEK/TLR5 assay

HEK/TLR5 cells were maintained in DMEM supplemented with 10% FCS, 10 µg/ml Blasticidin and 100 µg/ml Normocin. Monolayers of HEK/TLR4 and HEK/TLR5 cells were preincubated with 5-fold diluted bacterial supernatant for 30 min and subsequently stimulated with flagellin for 6 h at 37°C. Cell culture supernatant was harvested and stored at −20°C for analysis. Samples were diluted in HPE buffer and IL-8 concentrations were determined by ELISA following manufacturer's protocol, using a standard curve. For some experiments relative IL-8 amounts are expressed as OD at 450 nm.

To measure NF-κB activation, HEK/TLR4 and HEK/TLR5 cells were transiently transfected 2–3 days before stimulation with a NF-κB reporter plasmid pHIV-CAT [43] (AIDS Research and Reference Reagent Program, Division of AIDS, NIAID, NIH). Transfected cells were stimulated with LPS or flagellin for 5 h at 37°C. Cells were lysed with lysis buffer/substrate (Promega) according to manufacturer's protocol and chemiluminescence was measured using a Centro LB 960 microplate luminometer (Berthold). NF-κB activation is expressed as stimulation index, which represents the ratio between stimulated versus control cells.

### Neutrophil assay

Flagellin was incubated with 1 µg/ml AprA in the presence of 10 µg/ml polymyxin B (PMB) (Sigma) for 30 min at 37°C. Neutrophils (1.25×10^5^/well) were stimulated with AprA-treated flagellin for 16 h at 37°C. Cell culture supernatant was harvested and stored at −20°C for analysis by IL-8 ELISA.

### Isolation and purification of AprA

*P. aeruginosa* (clinical isolate) was cultured overnight in IMDM under constant agitation and supernatant was collected by centrifugation and filtration. Supernatant was applied on a Q sepharose XL column and eluted with PBS +2 M NaCl pH 7.4 using an Akta FPLC system (GE Healthcare). Active fractions were pooled and concentrated by lyphophilization and resuspended in PBS before gel filtration on a Superdex 75 column (GE Healthcare). Subsequently, active fractions were precipitated with trichloroacetic acid and separated by 12.5% SDS-PAGE gels and stained with silver. Proteins of interest were identified by mass-spectrometry (Alphalyse).

Recombinant AprA was produced in *E. coli* using two plasmids: i) pAG302 (kindly provided by A. de Groot) contains the *aprA* and *aprI* genes under control of the tac promoter on vector pUR6500, a derivative of pMMB67EH containing a kanamycin-resistance cassette; ii) pJF1 (kindly provided by J. Folders) containing the a*prD,* a*prE, and* a*prF* genes under control of the *lac* promoter on vector pBBR1MCS, which contains a chloramphenicol-resistance gene and is necessary for secretion of AprA. The two plasmids were used to transform *E. coli* BL21 and clones resistant to chloramphenicol and kanamycin were selected. Protein expression was induced by addition of 1 mM isopropyl β-D-1-thiogalactopyranoside (IPTG) for 3–4 h at 37°C. Supernatant was collected and diafiltrated against 20 mM phosphate buffer pH 7 using a proflux M-12 system (Millipore). AprA was applied on a Q sepharose XL column and eluted with 20 mM phosphate-buffered 2 M NaCl pH 7. Fractions containing AprA were concentrated using a 3 kD filter (Millipore) and applied on a Superdex 75 column.

For His-tagged AprA, the gene *aprA* (without the nine residues propeptide) was amplified from genomic PAO1 DNA using a forward and reverse primer with incorporated 5′BamH1 and 3′Not1, respectively. The PCR product was ligated in a modified pET302 vector (Invitrogen) encoding an N-terminal 6x His-tag and transformed in *E. coli* Top10F' cells (Invitrogen). After sequence verification, the constructs were transformed in *E. coli* BL21 and His-tagged proteins was purified under denaturing conditions according to manufacturer's instructions using a His trap column (GE Healthcare). Denatured AprA was diluted 50 times in 0.8 M L-arginine +50 mM Tris-HCl pH 9+1 mM CaCl_2_ at 4°C overnight [44]. After renaturation, AprA was concentrated on a Amicon 30 kD spin column (Millipore) and washed two times with 50 mM HEPES pH 7.8+1 mM CaCl_2_+0.1 mM ZnCl_2_. Purity was examined with SDS-PAGE and Coomassie staining.

### AprI isolation

The gene *aprI* without signal sequence of *P. aeruginosa* strain PAO1 with *Xba*1 restriction site, 6x His-tag and enterokinase cleavage site fused to the 5′-end and a 3′ *Eco*R1 restriction site was synthesized by BaseClear. The *aprI* construct was ligated into a pRSETB vector and used to transform *E. coli* Top10F' cells. Protein expression was performed in *E. coli* BL21 and alkaline protease inhibitor was purified under denaturing conditions according to manufacturer's instructions using a His trap FF column (GE Healthcare). Purified denatured protein was diluted 10-fold in PBS and concentrated on a His trap column. Purity of AprI was assessed by SDS-PAGE and the protein was dialyzed against PBS.

### Recombinant flagellin isolation

Constructs for recombinant *S.* Typhimurium and *P. aeruginosa* flagellin were generated by an overhang extension polymerase chain reaction (PCR) as described previously [40]. Briefly, genes *fliC* of *S.* Typhimurium (clinical isolate) and flagellin type B of *P. aeruginosa* strain PAO25 were cloned directly downstream of the 6x His-tag and enterokinase cleavage site of the pRSETB vector (Invitrogen). PCRs were performed using VentR DNA polymerase (New England Bio Labs). After verification of the sequence the vector was used to transform *E. coli* BL21 and the protein expression was performed as described for AprA. His-tagged flagellin was isolated by lysing bacteria with cellytic B according to manufacturer's instructions (Sigma) supplemented with DNAse/RNAse and protease inhibitor cocktail (Roche) followed by purification with a His trap FF column. The protein was eluted with 0.5 M imidazole in PBS and dialyzed against PBS. Purity was assessed by SDS-PAGE with Coomassie staining.

### Isolation of native flagellar filaments from *P. aeruginosa*

*P. aeruginosa* strain PAO25 (flagellin type B) and a clinical isolate (flagellin type A) were grown overnight in Luria-Bertani broth and bacteria were pelleted by centrifugation. Pellets were resuspended in PBS and flagella were sheared from bacteria by blending, followed by centrifugation at 8,000 g for 15 min to pellet the bacteria. Flagella were collected from the supernatant by centrifugation at 100,000 g for 60 min. The pellets obtained were resuspended in PBS and purity was examined by SDS-PAGE. Isolated flagella were heated at 70°C for 20 min for depolymerization.

### *P. aeruginosa* transposon mutants

*P. aeruginosa* mutants *aprA1* (3969) and *aprA2* (16254) were obtained from the *P. aeruginosa* PAO1 transposon mutant library [45] (University of Washington). Transposon insertions in the *aprA* mutant strains were confirmed by PCR. Bacteria were grown overnight in IMDM and supernatant was harvested by centrifugation and filtration. Where indicated, 3 µg/ml recombinant AprA or 10 µg/ml AprI was added to the culture medium before inoculation with the strains. HEK/TLR5 cells were stimulated with dilutions of these overnight culture supernatants and IL-8 release was measured by ELISA. For neutrophil stimulation bacterial supernatant was incubated with 10 µg/ml PMB for 30 min at 37°C. Neutrophils were stimulated with untreated and PMB-treated bacterial supernatant for 16 h at 37°C. Cell culture supernatant was harvested and IL-8 concentration was determined by ELISA.

### Flagellin and flg22 degradation

Recombinant AprA, AprI and flagellin of *P. aeruginosa* or *S.* Typhimurium in PBS were incubated for 1 h (unless specified otherwise) at 37°C. Cleavage products were analyzed by SDS-PAGE and stained with Coomassie. AprA was inhibited by preincubation with 100 mM EDTA or AprI before addition of flagellin. Flg22 was incubated with 1 µg/ml AprA for 1 h at 37°C. The sample was spotted on a NP-20 array (Biorad) and analyzed using a ProteinChip SELDI reader (Biorad).

### AprA detection

The presence of AprA in supernatants of *P. aeruginosa* mutants was checked by Western blotting using a polyclonal rabbit AprA antiserum (generously provided by R. Voulhoux). Recombinant AprA or bacterial supernatant was separated with SDS-PAGE. Proteins were transferred to an Immobilon-P membrane (Millipore) and blocked with 4% skimmed milk in PBS + 0.05% Tween. Subsequently blots were incubated with 1/500 diluted rabbit AprA antiserum followed by HRP-conjugated goat-anti-rabbit IgG (Biorad) and bands were visualized by enhanced chemiluminescence (Amersham).

### *Arabidopsis* callose deposition assay

Seeds of *A. thaliana* ecotype Landsberg *erecta* (La-*er*) were vapor face sterilized and sown on Murashige-Skoog (MS; Sigma) medium containing 0.6% Plant Agar (Duchefa) and 1% (w/v) sucrose (Sigma). After a two-day vernalization period at 4°C, plates were transferred to growth chambers with an 8 h day (200 µEm^−2^.sec^−1^ at 24°C) and 16 h night (20°C) cycle for seven days. Three seedlings were transferred to a single well containing 1 ml MS containing 1% (w/v) sucrose and the components required for the treatments as indicated in the text. After 24 h the medium was replaced by 1 ml 96% EtOH followed by incubation overnight for removal of chlorophyll. The next day, decolorized seedlings were washed in 0.07 M phosphate buffer (pH 9) and subsequently incubated with the same buffer containing 0.01% aniline blue (water blue; Merck). Samples were placed in the dark for a period of 20 h at RT. Microscopic slides were prepared in a matrix of fresh aniline blue. Observations were performed with a fluorescence microscope (Olympus Ax70 with Olympus U-RFL-T) with UV filter (bandpass 340 to 380 nm, long-path 425 nm) [46].

### *Arabidopsis* growth assay

Ten vapor-face sterilized seeds of *A. thaliana* (La-*er*) were transferred to a well of 24-well plates containing 1 ml MS medium with 1% (w/v) sucrose and treatment-specific components. Seeds were vernalized by putting the 24-well plates at 4°C for two days. Subsequently, plates were transferred to growth chambers with an 8-h day (200 µEm^−2^.sec^−1^ at 24°C) and 16 h night (20°C) cycle. Differences in growth rate were monitored after 7–10 days by photography.

### Stomatal closure assay

Wild-type *A. thaliana* Col-0 plants were grown for 5 weeks in an autoclaved mixture of sand/potting soil in a growth chamber with a 9 h day (200 µE m^−2^ s^−1^ and 24°C) and 15 h night (20°C) cycle and 70% relative humidity as described [47]. Wild-type *P. aeruginosa* PAO1 *aprA* mutant strains were grown overnight in liquid Kings Medium B at 28°C. The leaves of 5-week-old plants were dipped for 2 seconds in a solution of 10 mM MgSO_4_ and 0.015% (v/v) Silwet containing 2.5·10^7^ cfu/ml of *P. aeruginosa* bacteria. The epidermis of two leaves were peeled off before treatment (t = 0) and 5, 10, 20 and 40 min after treatment and immediately observed under a Zeiss Axioskop2 microscope (400x). Pictures were taken at 10–15 random regions of the leaves. Stomatal aperture was then measured using the software package ImageJ.

### Accession numbers

Swiss-prot accession numbers: *P. aeruginosa* AprA (Q03023), AprI (Q03026), flagellin type B (P72151) and flagellin from *S.* Typhimurium (P06179).

## Supporting Information

Figure S1 **Fractionated** ***P. aeruginosa*** **supernatant does not inhibit IL-8 production of HEK/TLR4 cells.** *P. aeruginosa* supernatant was purified by ion-exchange chromatography and concentrated before gel filtration. Proteins were separated by size (Superdex 75) and 0.5 ml fractions were collected. HEK/TLR4 (white bars) and HEK/TLR5 (black bars) cells were pretreated with gel filtration fractions B5-B12 (10-fold diluted) for 30 minutes and subsequently challenged with 1 ng/ml LPS or flagellin, respectively. After 6 h stimulation cell culture supernatant was harvested and IL-8 concentration was determined by ELISA.
(TIF)

Figure S2 **Identification of TLR5 inhibitory protein.** *P. aeruginosa* supernatant was separated by anion exchange (Q sepharose XL) and size exclusion chromatography (Superdex 75). (A) HEK/TLR5 cells were incubated with fractions after size exclusion chromatography for 30 min at 37°C, and subsequently challenged with 1 ng/ml flagellin for 6 h at 37°C. IL-8 levels were determined in the cell culture supernatant by ELISA. (B) Fractions were analyzed with SDS-PAGE and Coomassie staining.
(TIF)

Figure S3 **EDTA blocks AprA-mediated flagellin cleavage.** Flagellin (250 μg/ml) and AprA (1 μg/ml) were incubated in the presence of 10 mM or 100 mM EDTA for 60 min at 37°C. Flagellin degradation was analyzed by SDS-PAGE and Coomassie staining.
(TIF)

Figure S4 **Dose-dependent inhibition of AprA by AprI.** HEK/TLR5 cells were challenged with 1 ng/ml *S.* Typhimurium flagellin in the presence of an increasing concentration AprA premixed with AprI at 0, 20, 61, 200 or 610 nM. After six hours IL-8 production was measured by ELISA.
(TIF)

Figure S5 **AprA prevents flagellin recognition in** ***Arabidopsis*** **.** *A. thaliana* La-*er* seedlings were incubated or not with 500 nM flg22 or *P. aeruginosa* flagellin that was preincubated with 3 µg/ml AprA when indicated. After treatment, seedlings were grown axenically for 10 days in MS medium and subsequently photographed. In the 3^rd^ row panels AprI was added before AprA treatment and in the bottom panels post AprA treatment (p.t.) of flagellin.
(TIF)

Figure S6 **SELDI-TOF analysis of flg22 cleavage by AprA.** Flg22 (50 μM) was incubated with buffer (upper panel) or 3 μg/ml AprA (bottom panel) for 1 h at 37°C. For SELDI-TOF analysis untreated and AprA-treated peptide was diluted 250 times (200 nM) and spotted on a NP-20 array. Results between 500 Da and 2500 Da are shown, the 2274 Da peak represents flg22.
(TIF)
