# Supplementary figures and images for: Pseudomonas Evades Immune Recognition of Flagellin in Both Mammals and Plants
Source: PLoS Pathog. 2011 Aug 25;7(8):e1002206. doi: 10.1371/journal.ppat.1002206 (PMC3161968; doi:10.1371/journal.ppat.1002206)

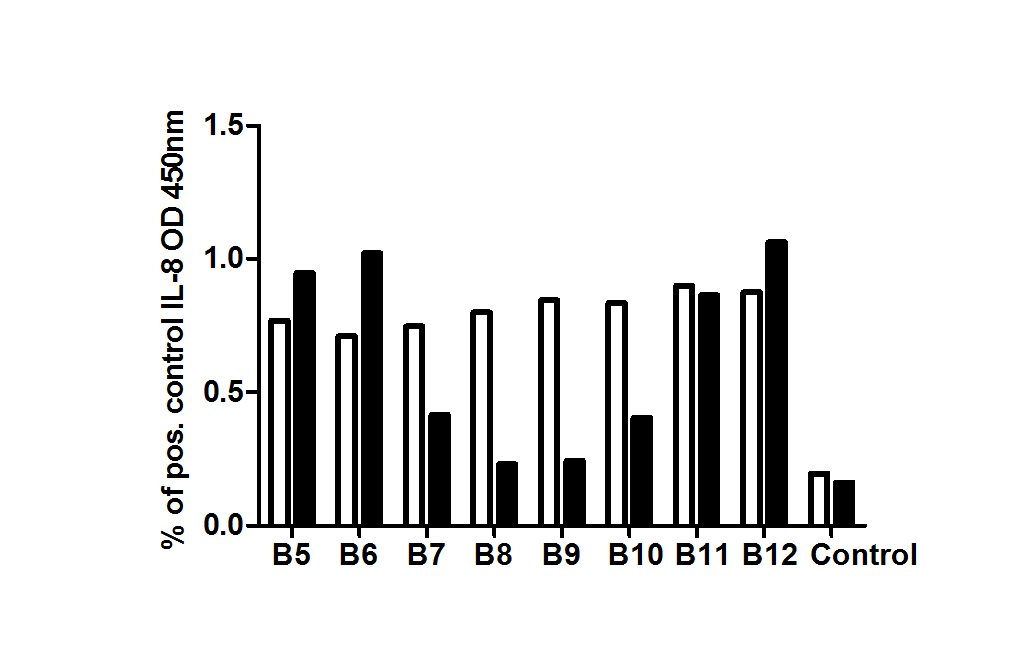

Supplement: Figure S1 — Fractionated P. aeruginosa supernatant does not inhibit IL-8 production of HEK/TLR4 cells. P. aeruginosa supernatant was purified by ion-exchange chromatography and concentrated before gel filtration. Proteins were separated by size (Superdex 75) and 0.5 ml fractions were collected. HEK/TLR4 (white bars) and HEK/TLR5 (black bars) cells were pretreated with gel filtration fractions B5-B12 (10-fold diluted) for 30 minutes and subsequently challenged with 1 ng/ml LPS or flagellin, respectively. After 6 h stimulation cell culture supernatant was harvested and IL-8 concentration was determined by ELISA. (TIF) [file ppat.1002206.s001.tif]

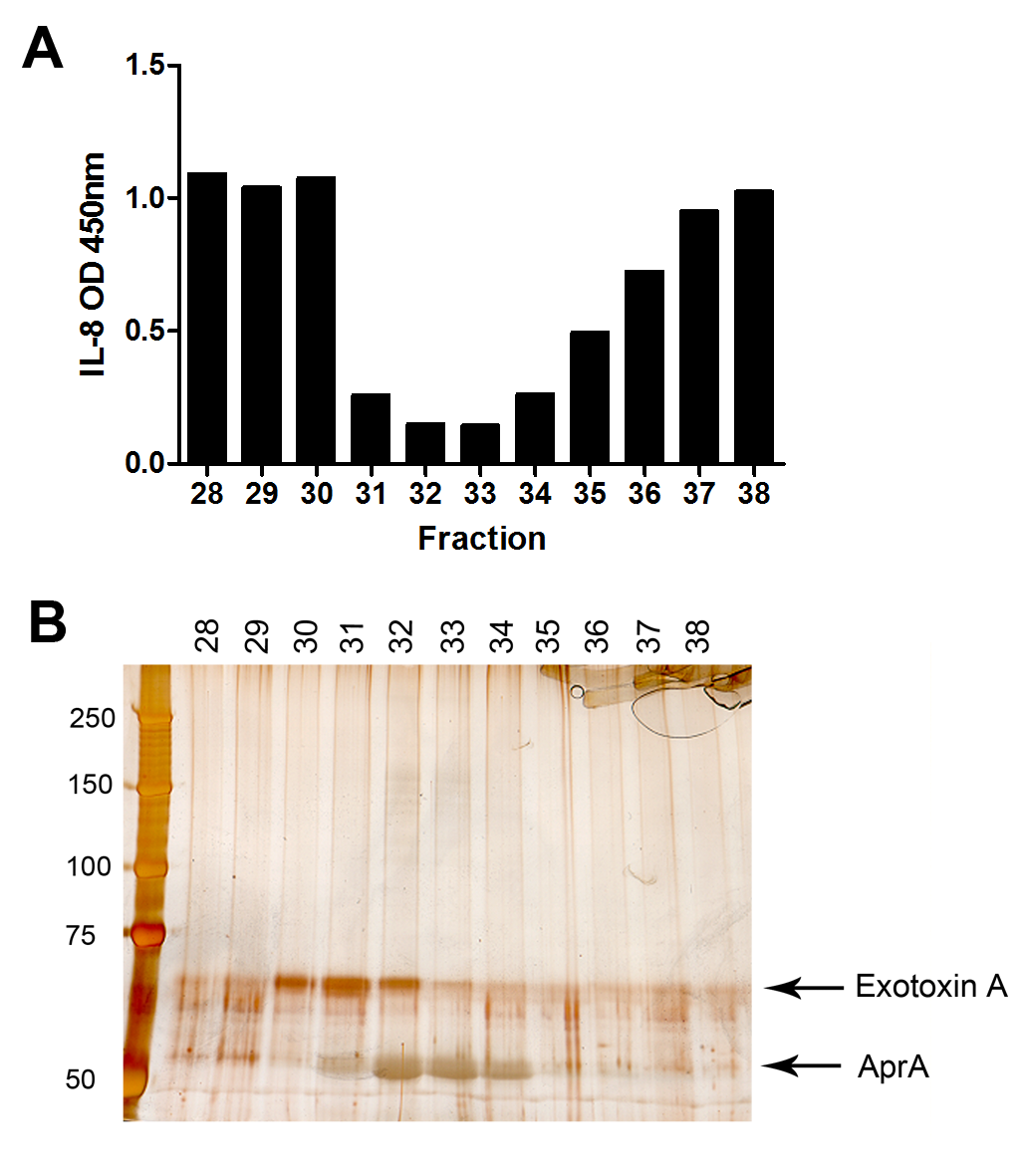

Supplement: Figure S2 — Identification of TLR5 inhibitory protein. P. aeruginosa supernatant was separated by anion exchange (Q sepharose XL) and size exclusion chromatography (Superdex 75). (A) HEK/TLR5 cells were incubated with fractions after size exclusion chromatography for 30 min at 37°C, and subsequently challenged with 1 ng/ml flagellin for 6 h at 37°C. IL-8 levels were determined in the cell culture supernatant by ELISA. (B) Fractions were analyzed with SDS-PAGE and Coomassie staining. (TIF) [file ppat.1002206.s002.tif]

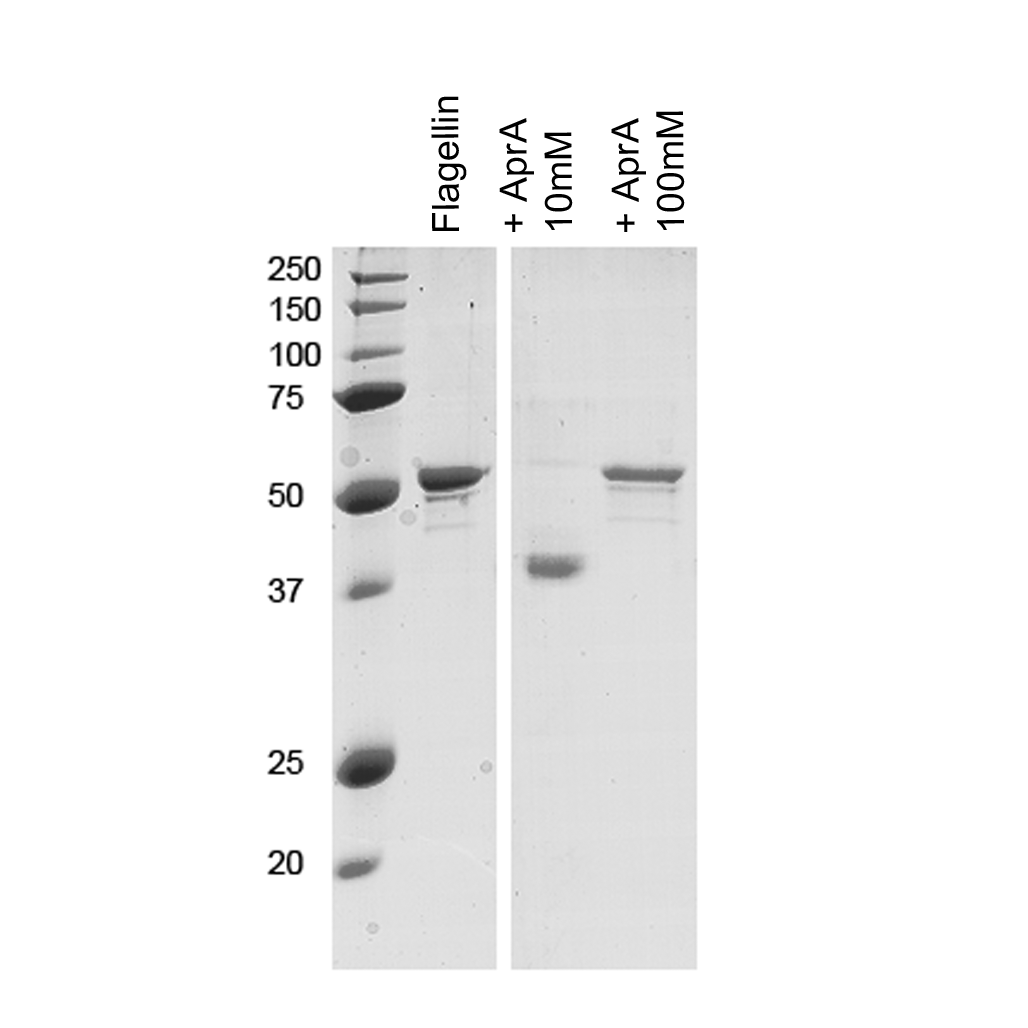

Supplement: Figure S3 — EDTA blocks AprA-mediated flagellin cleavage. Flagellin (250 μg/ml) and AprA (1 μg/ml) were incubated in the presence of 10 mM or 100 mM EDTA for 60 min at 37°C. Flagellin degradation was analyzed by SDS-PAGE and Coomassie staining. (TIF) [file ppat.1002206.s003.tif]

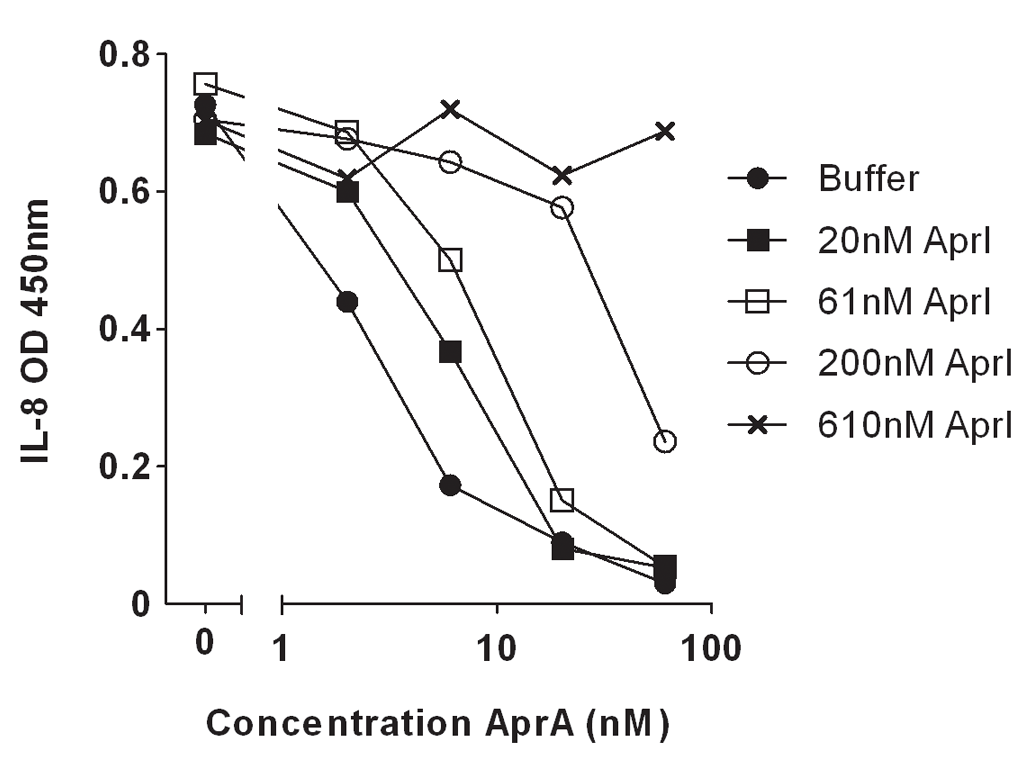

Supplement: Figure S4 — Dose-dependent inhibition of AprA by AprI. HEK/TLR5 cells were challenged with 1 ng/ml S. Typhimurium flagellin in the presence of an increasing concentration AprA premixed with AprI at 0, 20, 61, 200 or 610 nM. After six hours IL-8 production was measured by ELISA. (TIF) [file ppat.1002206.s004.tif]

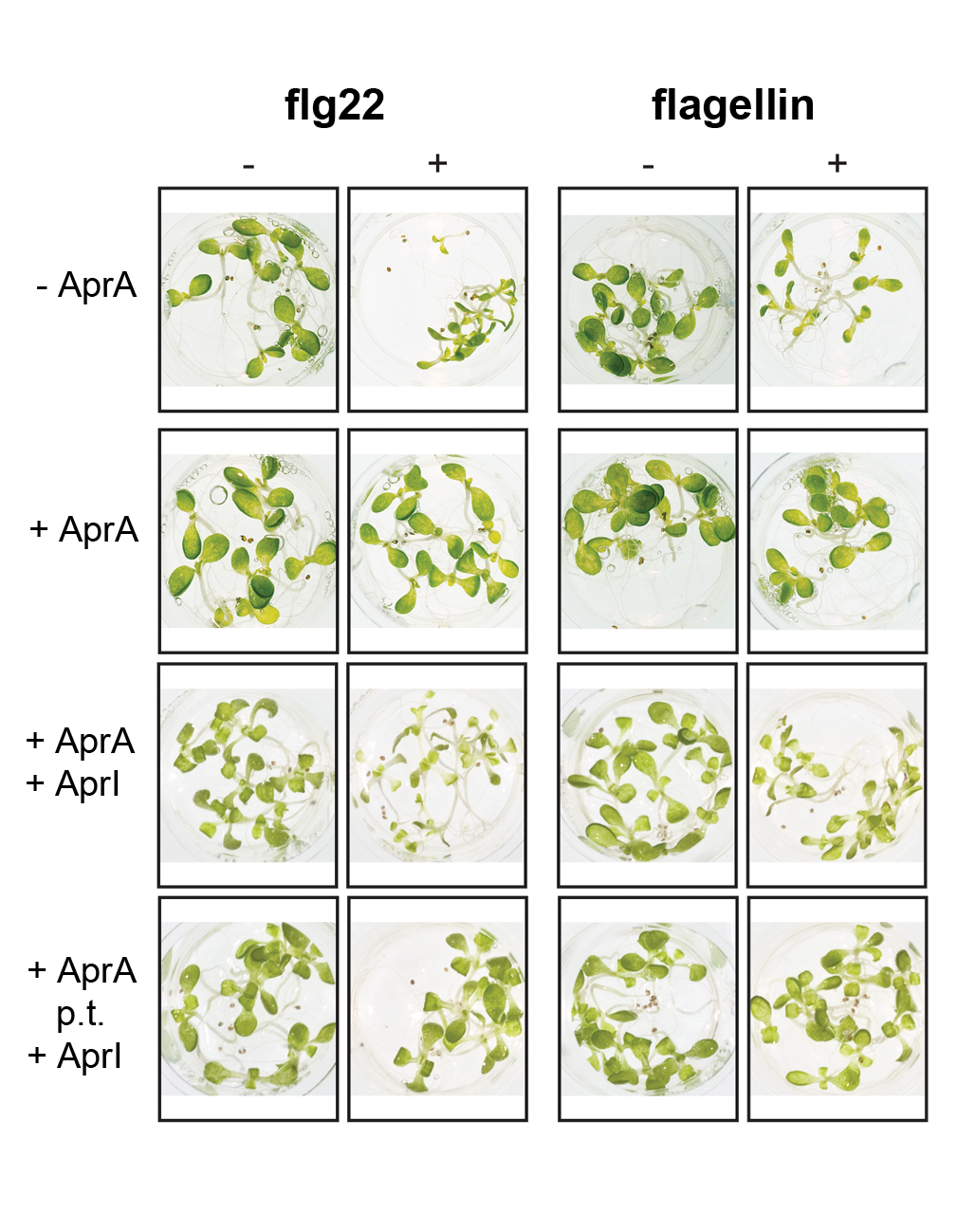

Supplement: Figure S5 — AprA prevents flagellin recognition in Arabidopsis . A. thaliana La-er seedlings were incubated or not with 500 nM flg22 or P. aeruginosa flagellin that was preincubated with 3 µg/ml AprA when indicated. After treatment, seedlings were grown axenically for 10 days in MS medium and subsequently photographed. In the 3rd row panels AprI was added before AprA treatment and in the bottom panels post AprA treatment (p.t.) of flagellin. (TIF) [file ppat.1002206.s005.tif]

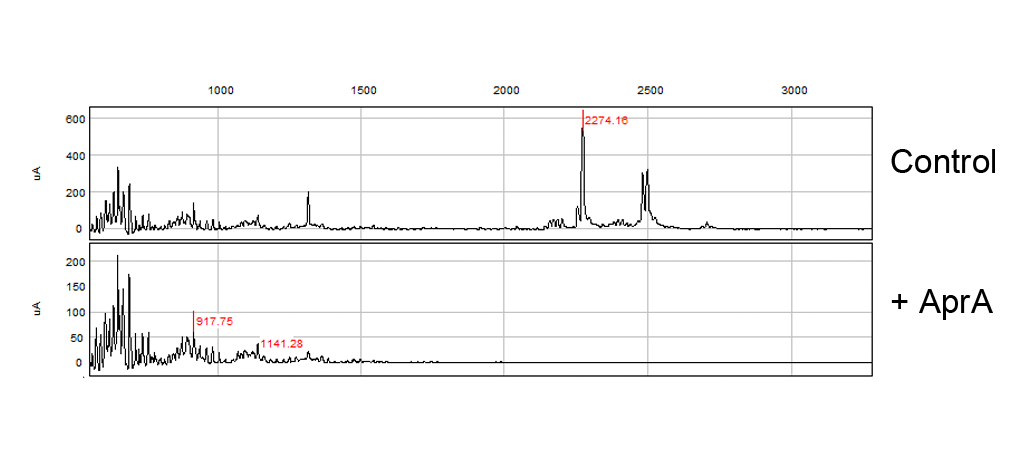

Supplement: Figure S6 — SELDI-TOF analysis of flg22 cleavage by AprA. Flg22 (50 μM) was incubated with buffer (upper panel) or 3 μg/ml AprA (bottom panel) for 1 h at 37°C. For SELDI-TOF analysis untreated and AprA-treated peptide was diluted 250 times (200 nM) and spotted on a NP-20 array. Results between 500 Da and 2500 Da are shown, the 2274 Da peak represents flg22. (TIF) [file ppat.1002206.s006.tif]
